# Wearable Sensor Use for Assessing Standing Balance and Walking Stability in People with Parkinson’s Disease: A Systematic Review

**DOI:** 10.1371/journal.pone.0123705

**Published:** 2015-04-20

**Authors:** Ryan P. Hubble, Geraldine A. Naughton, Peter A. Silburn, Michael H. Cole

**Affiliations:** 1 School of Exercise Science, Australian Catholic University, Brisbane, Queensland, Australia; 2 School of Exercise Science, Australian Catholic University, Melbourne, Victoria, Australia; 3 The University of Queensland, Centre for Clinical Research, Brisbane, Queensland, Australia; University of Tuebingen, GERMANY

## Abstract

**Background:**

Postural instability and gait disability threaten the independence and well-being of people with Parkinson’s disease and increase the risk of falls and fall-related injuries. Prospective research has shown that commonly-used clinical assessments of balance and walking lack the sensitivity to accurately and consistently identify those people with Parkinson’s disease who are at a higher risk of falling. Wearable sensors provide a portable and affordable alternative for researchers and clinicians who are seeking to objectively assess movements and falls risk in the clinical setting. However, no consensus currently exists on the optimal placements for sensors and the best outcome measures to use for assessing standing balance and walking stability in Parkinson’s disease patients. Hence, this systematic review aimed to examine the available literature to establish the best sensor types, locations and outcomes to assess standing balance and walking stability in this population.

**Methods:**

Papers listed in three electronic databases were searched by title and abstract to identify articles measuring standing balance or walking stability with any kind of wearable sensor among adults diagnosed with PD. To be eligible for inclusion, papers were required to be full-text articles published in English between January 1994 and December 2014 that assessed measures of standing balance or walking stability with wearable sensors in people with PD. Articles were excluded if they; i) did not use any form of wearable sensor to measure variables associated with standing balance or walking stability; ii) did not include a control group or control condition; iii) were an abstract and/or included in the proceedings of a conference; or iv) were a review article or case study. The targeted search of the three electronic databases identified 340 articles that were potentially eligible for inclusion, but following title, abstract and full-text review only 26 articles were deemed to meet the inclusion criteria. Included articles were assessed for methodological quality and relevant data from the papers were extracted and synthesized.

**Results:**

Quality assessment of these included articles indicated that 31% were of low methodological quality, while 58% were of moderate methodological quality and 11% were of high methodological quality. All studies adopted a cross-sectional design and used a variety of sensor types and outcome measures to assess standing balance or walking stability in people with Parkinson’s disease. Despite the typically low to moderate methodological quality, 81% of the studies reported differences in sensor-based measures of standing balance or walking stability between different groups of Parkinson’s disease patients and/or healthy controls.

**Conclusion:**

These data support the use of wearable sensors for detecting differences in standing balance and walking stability between people with PD and controls. Further high-quality research is needed to better understand the utility of wearable sensors for the early identification of Parkinson’s disease symptoms and for assessing falls risk in this population.

**PROSPERO Registration:**

CRD42014010838

## Introduction

Parkinson’s disease (PD) is an age-related neurodegenerative disorder that results from the loss of neurons within the basal ganglia that produce dopamine, an important neurotransmitter involved in the regulation of movement. As medical advances have extended the life expectancy of the average person, clinical and experimental methods need to progress as well in order to improve the management of the symptoms associated with the disease. It is well understood that deficits in balance and gait are common and disabling features of PD that significantly increase an individual’s risk of falling [1]. Subsequently, many clinical assessments have been developed to evaluate these symptoms in this population. The most common assessments include the Berg Balance Scale [2, 3], the Tinetti Gait and Balance assessment [2], the Timed up and Go test [2, 4] and the postural instability and gait disability (PIGD) score derived from the Unified Parkinson’s Disease Rating Scale (UPDRS) [2, 5]. These assessments are suited to clinical settings because they require little equipment to conduct and provide almost immediate outcomes that can be reported to the patient. However, prospective research shows these tests have poor sensitivity and specificity for identifying prospective fallers in the PD population [2] and may not be sufficiently sensitive to detect changes in balance and walking in people with PD who have mild to moderate disease severity [6–9].

Given the inherent short-comings of the aforementioned clinical tests, previous research has sought to improve the objectivity of these measures to enhance their ability to track symptom progression and evaluate patient risk. Camera-based three-dimensional motion analysis systems have been commonly used in laboratory settings to examine the walking patterns of people with PD [10–12]. However, the methods associated with these assessments are often time-consuming and require specific expertise and expensive motion capture systems that are impractical for smaller clinical spaces. Wearable sensors, such as accelerometers or inertial measurement units (IMUs), offer a more portable, flexible and moderately-priced alternative to camera-based motion analysis systems. Moreover, wearable sensors do not require excessive space for normal operation and outcome measures can be output almost immediately without the need for significant post-processing procedures. Given these strengths, research has recently sought to improve the sensitivity of clinical assessments, such as the Timed Up and Go test, by incorporating accelerometers or IMUs to provide continuous measures of walking [13–17]. The results of this research demonstrated that it was possible to detect differences in the performances of people with PD compared with controls by instrumenting the Timed Up and Go test with a wearable sensor [13–17].

Wearable sensors have recently shown good test-retest reliability for assessing individuals with PD, particularly for acceleration-based measures calculated in the time domain (e.g. jerk; the first time derivative of acceleration) [13]. Furthermore, a growing body of literature supports the use of wearable sensors to assess standing balance or walking for; i) people with PD and controls [13, 14, 18–29]; ii) PD fallers and non-fallers [30, 31]; iii) people with different PD sub-types [17, 32–35]; iv) carriers and non-carriers of the LRRK2 gene [36]; and v) people at high risk of developing PD (HRPD) [37, 38]. Results from these studies demonstrate that outcomes derived from wearable sensors are effective for detecting differences in standing balance between HRPD patients, people with PD and controls [38]. When combined in a logistic regression model, it was evident that outcome measures derived from wearable sensors can discriminate HRPD patients from controls using an instrumented functional reach test [37]. Furthermore, three-dimensional accelerometers positioned on the head, trunk or pelvis, have highlighted less rhythmic walking patterns for people with PD who retrospectively reported falling than patients without falls [30, 31]. Collectively, these results suggest that wearable sensors may not only be useful for evaluating changes in a patient’s balance or gait patterns, but may also offer a means of screening individuals for various risk factors associated with PD or falls. Nevertheless, scientifically-rigorous prospective research is needed before stronger recommendations can be provided regarding the use of these devices as predictive instruments for clinical populations.

Despite the expanding body of evidence to support the use of wearable sensors for assessing function in people with PD, it is important to recognise that this area of science is still developing. Furthermore, the adoption of such varying methodological approaches in the existing literature makes it difficult to determine which type of sensor is the best to use and which placements and outcome measures are optimal to maximise the utility of these devices. As such, it was the purpose of this systematic review to examine the available literature that utilised wearable sensors to measure standing and walking balance in people with PD and provide a summary of the best sensor types, locations and outcomes based on a consensus of the literature.

## Methods

This review was registered with the International Prospective Register of Systematic Reviews on September 3, 2014 (PROSPERO Registration: CRD42014010838). The search strategy and study protocol are available at http://www.crd.york.ac.uk/PROSPEROFILES/10838_STRATEGY_20141106.pdf.

### Search Strategy

An electronic database search of titles and abstracts was performed in January 2015 using PubMed, EMBASE and the Cochrane Library to identify articles measuring standing balance and walking stability with any kind of wearable sensor among adults diagnosed with PD. The following terms were used for the literature search: ‘Parkinson’, ‘Parkinson’s’, ‘walk’, ‘gait’, ‘balance’, ‘stability’, ‘sensor’, ‘gyroscope’, ‘inertial’, ‘acceleration’ and ‘accelerometer’. Specifically, papers that were included in this review were required to have the term ‘Parkinson or Parkinson’s’ AND (‘walk’ OR ‘gait’ OR ‘balance’ OR ‘stability’) AND (‘sensor’ OR ‘gyroscope’ OR ‘inertial’ OR ‘accelerometer’ OR ‘acceleration’) located within the title and/or abstract. In addition to the systematic electronic database search, a targeted search of the bibliographies of relevant articles was also performed to identify any additional studies for inclusion. The research protocol for this systematic review is included as Supporting Information and outlines the procedures followed and the exact search strategy used for this study (S1 File).

### Selection Criteria

**Fig 1:**
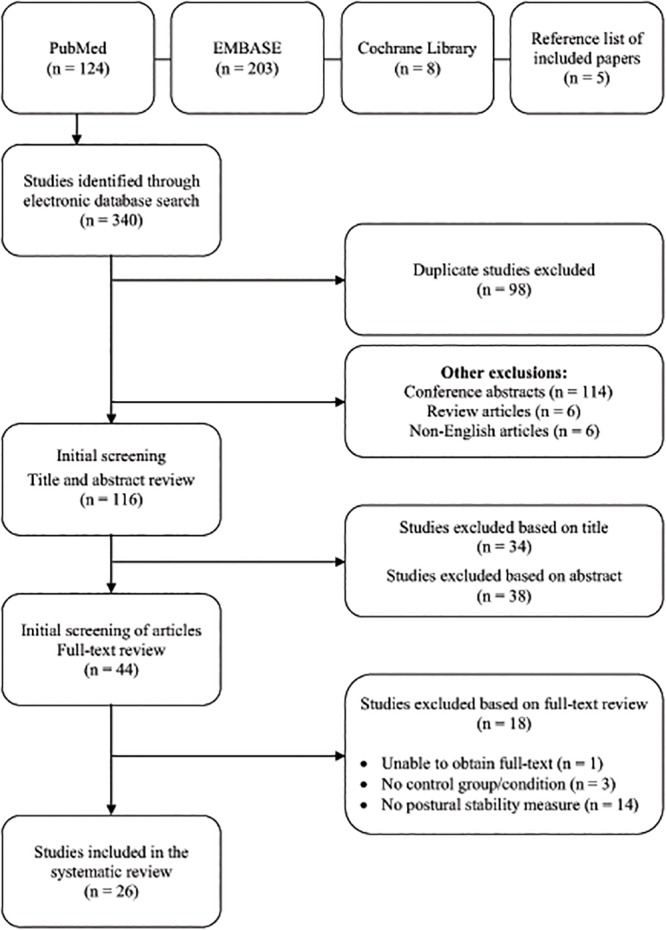
Flow diagram outlining the progression of the study’s systematic search strategy and review process, which led to the identification of the articles included in the review.

Only original, full-text articles published in English between January 1994 and December 2014 that assessed standing balance or walking stability with wearable sensors in people with PD were included in this review. Articles were excluded if they; i) did not use any form of wearable sensor to measure variables associated with standing balance or walking stability; ii) did not include a control group or control condition; iii) were an abstract and/or included in the proceedings of a conference; or iv) were a review article or case study. All studies that met the inclusion criteria were considered for review, irrespective of their research design (cross-sectional, randomised controlled trial, etc). After the initial literature search was completed, two assessors (RPH, MHC) independently screened each of the papers based on their title and abstract and made a decision on the suitability of the paper for inclusion in the review. Once both reviewers had completed this process, any and all discrepancies between the two assessments were discussed until a consensus was reached regarding each paper. Full-text articles were retrieved for all of the papers selected for inclusion based on the title and abstract review process and the full-text of these articles was reviewed for suitability by one assessor (RPH). A flow diagram illustrating the study selection process is provided in Fig 1.

### Data Extraction and Quality Assessment

Upon selection of the articles for inclusion, one assessor (RPH) extracted and collated information concerning the type and number of participants, their mean age, disease duration and symptom severity, as well as the type and location of the wearable sensor(s) used and the major findings of each study (Table 1). The included studies presented a range of outcomes that sought to gain a better insight into the deficits of standing balance and walking stability evident in people with PD and these included; i) the root mean square (RMS) of segmental accelerations; ii) the harmonic ratio; iii) jerk (the first derivative of acceleration); iv) step or stride variability; v) step or stride regularity/symmetry; and vi) other less commonly-used measures of stability.

**Table 1:** Summarises the major characteristics of the research design, analyses and outcomes for the studies that met the inclusion criteria for this review.

Article	Experimental Groups N (Mean Age ± SD)	Disease Severity	Disease Duration (Years)	Sensor Type (Placement)	Postural Stability Measures	Modality	Findings
Baston 2014 ^[^ ^24^ ^]^	PD = 5 (62.0±6.0) PSP = 7 (68.0±5.0) Control = 7 (68.0±7.0)	***UPDRS III*** PD = 34.0±14.0	Not Reported	Inertial Sensor Freq: 128 Hz L5 Shank	RMS acceleration *Anteroposterior (AP)*	Dynamic Posturography	No significant difference between PD and controls for AP acceleration during all conditions of the Sensory Organisation Test (SOT). PD had reduced AP accelerations for conditions 4 and 5 of the SOT compared with the PSP group.
Fazio 2012 ^[^ ^18^ ^]^	PD = 17 (60–85) Ataxia = 24 (20–85) Control = 24 (20–85)	***UPDRS III*** PD = 22.5±3.6	Not Reported	3D Accelerometer Freq: 20 Hz Sternum Front pelvis Back pelvis	RMS acceleration *For sum of sternum accelerations For sum of front pelvis accelerations For sum of back pelvis accelerations* RMS Jerk *For sum of sternum accelerations*	Gait	PD patients had lower Jerk scores compared with controls, but were not significantly different to ataxic patients. PD had significantly lower RMS accelerations for the sternum and two pelvis locations compared with the ataxic and control participants.
Gago 2014 ^[^ ^32^ ^]^	IPD = 10 (73 [61–79]) VPD = 5 (77 [63–84])	***MDS-UPDRS III*** IPD = 30 [15–53] VPD = 44 [33–57]	IPD 6.0 [5.0–10.0] VPD 5.0 [3.0–9.0]	3D Accelerometer Freq: 113 Hz Lower back	Length of sway Maximum sway distance Mean sway distance Maximum linear velocity	Quiet Stance	Idiopathic PD (IPD) patients had significantly increased length and maximum distance of sway during normal stance while on medication. Sway length and maximum distance was also greater for the IPD group when eyes were closed compared with open during the Romberg test off medication. Compared with the IPD patients, vascular PD (VPD) patients had increased mean distance of sway during normal stance and greater maximal distance of sway compared with the IDP patients during the Romberg test with eyes closed off medication.
Hasmann 2014 ^[^ ^37^ ^]^	PD = 13 (65.0±9.4) HRPD = 31 (62.6±5.0) Control = 13 (63.9±7.3)	***UPDRS III*** PD = 26.8±11.0 HRPD = 3.0±3.0 Control = 0.2±0.6	PD 4.5±2.8	3D Accelerometer Freq: Not reported Lower back	Mean acceleration *Anteroposterior (AP) Mediolateral (ML)* Jerk *Anteroposterior (AP) Mediolateral (ML)*	Functional Reach	Compared with controls, PD had increased mean acceleration in the AP and ML directions, but the groups did not differ significantly with respect to AP or ML Jerk scores.
Herman 2014 ^[^ ^17^ ^]^	PD PIGD = 31 (65.0±7.7) PD TD = 32 (64.6±11.6)	***UPDRS III—OFF*** PIGD = 38.7±10.5 TD = 39.5±12.5 ***UPDRS III—ON*** PIGD = 33.3±10.0 TD = 33.4±11.6	PIGD 5.7±3.7 TD 5.4±3.2	3D Accelerometer Freq: 100 Hz Lower back	Harmonic ratio (HR) *Anteroposterior (AP) Mediolateral (ML) Vertical (VT)* Stride regularity Stride timing variability	Gait	For usual walking, PIGD patients had reduced stride regularity and reduced vertical HRs compared with the TD group while off medication. Accelerometer-derived measures from a 3-day period of in-home activity monitoring revealed that the PIGD group had reduced stride regularity and lower harmonic ratios in both the AP and VT directions compared with the TD group.
Latt 2009 ^[^ ^30^ ^]^	***PD Fallers vs*. *Non-Fallers*:** Non-Faller = 33 (63.0±4.0) Faller = 33 (67.0±2.0) Control = 33 (67.0±4.0)	***Hoehn & Yahr*** Non-faller = 1 (1–1) Faller = 3 (3–4) ***UPDRS III*** Non-faller = 12.0±3.0 Faller = 21.0±3.0	PD NF 7.0±2.0 PD F 9.0±2.0	3D Accelerometer Freq: 200 Hz Head Sacrum	Harmonic ratio (HR) *Anteroposterior (AP) Mediolateral (ML) Vertical (VT)* RMS acceleration *Anteroposterior (AP) Mediolateral (ML) Vertical (VT)* Step timing variability	Gait	Compared with controls and PD non-fallers, fallers had increased step timing variability. With the exception of AP head accelerations, PD fallers had significantly reduced head and pelvis accelerations compared with non-fallers and controls. Controls had higher AP head accelerations compared with PD fallers, and PD non-fallers had lower ML accelerations for the pelvis than controls. PD fallers had lower AP and VT HRs for the head and lower AP, ML and VT HRs for the pelvis compared with non-fallers and controls. PD non-fallers had lower VT HRs for the head and pelvis and lower AP HRs for the head compared with controls. Non-fallers also had greater ML HRs for the head compared with fallers.
Lowry 2010 ^[^ ^39^ ^]^	PD = 7 (70.3±8.5)	***Hoehn & Yahr*** PD = 2.4±0.5	PD 6.2±4.7	3D Accelerometer Freq: 200 Hz L3	Harmonic Ratio (HR) *Anteroposterior (AP) Mediolateral (ML) Vertical (VT)*	Gait	Cognitive cueing (thinking “big step” during the swing phase) and verbal cueing (assessor saying “big step” during the swing phase) both improved AP HR compared with preferred gait (without cues).
Lowry 2009 ^[^ ^19^ ^]^	PD = 11 (68.0±7.7) Control = 11 (69.0±8.8)	***Hoehn & Yahr*** PD = 1.9±0.8	PD 5.2±4.0	3D Accelerometer Freq: 200 Hz L2	Harmonic Ratio (HR) *Anteroposterior (AP) Mediolateral (ML) Vertical (VT)* Stride timing variability Stride length variability	Gait	PD and controls did not differ significantly with respect to stride length variability, stride timing variability or AP, ML and VT HRs. After normalising these data to walking speed, PD patients had lower AP and ML HRs compared with controls.
Maetzler 2012 ^[^ ^38^ ^]^	PD = 12 (61.5±2.2) HRPD = 20 (61.9±1.5) Control = 14 (63.9±1.9)	***Hoehn & Yahr*** PD = 2.0±0.0 ***UPDRS III—OFF*** PD = 26.5±10.9 HRPD = 3.3±2.4 Control = 1.1±1.7	PD 4.3±2.6	Inertial Sensor Freq: 100 Hz L3/L4	RMS acceleration *Anteroposterior (AP) Mediolateral (ML)* Jerk *Anteroposterior (AP) Mediolateral (ML)* Frequency with 95% of signal (F95) *Anteroposterior (AP) Mediolateral (ML)* Mean sway velocity	Quiet Stance	The PD and control groups did not differ significantly for AP or ML RMS accelerations or Jerk scores, even when vision was occluded and/or somatosensory feedback was reduced. However, the high risk of PD (HRPD) group had greater AP and ML RMS accelerations than PD patients and controls while standing on a foam surface with eyes closed and greater scores than PD when standing on a firm surface with eyes closed. The HRPD group also had greater AP and ML Jerk scores than the PD and controls group during the foam eyes closed task. Groups did not differ with respect to F95 or mean sway velocity.
Mancini 2011 ^[^ ^26^ ^]^	PD = 13 (60.4±8.5) Control = 12 (60.2±8.2)	***Hoehn & Yahr*** PD = 1.8±0.6 ***UPDRS III*** PD = 28.2±11.2	PD 14.3±6.9	Inertial Sensor Freq: 50 Hz L5	RMS Acceleration *Resultant of AP and ML* Jerk *Resultant of AP and ML* Frequency with 95% of signal (F95) *Resultant of AP and ML* Mean sway velocity	Quiet Stance	Compared with controls, the PD group had significantly greater RMS accelerations, Jerk scores and mean sway velocity measures while standing on a firm surface with eyes open, but not with eyes closed. Groups did not differ with respect to the F95 measure.
Mancini 2012 ^[^ ^13^ ^]^	***Study 1*** PD = 13 (60.4±8.5) Control = 12 (60.2±8.2) ***Study 2*** PD = 17 (67.1±7.3) Control = 17 (67.9±6.1)	***Study 1 UPDRS III*** PD = 28.1±11.2 ***Study 2 UPDRS III*** PD = 28.3±10.4	Not Reported	Inertial Sensor Freq: 50 Hz L5	RMS Acceleration *Resultant of AP and ML* Jerk *Resultant of AP and ML* Frequency with 95% of signal (F95) *Resultant of AP and ML* Mean sway velocity Length of sway Mean sway distance Sway area	Quiet Stance	Compared with controls, the PD group had significantly higher RMS accelerations, Jerk scores, sway distances and sway areas, but the groups did not differ with respect to the F95 measure, mean sway velocities or length of sway.
Mancini 2012 ^[^ ^25^ ^]^	PD = 13 (60.4±8.5) Control = 12 (60.2±8.2)	***Hoehn & Yahr*** PD = 1.8±0.2(SEM) ***UPDRS III*** PD = 26.6±3.5(SEM)	Not Reported	Inertial Sensor Freq: 50 Hz L5	RMS acceleration *Anteroposterior (AP) Mediolateral (ML)* Jerk *Anteroposterior (AP) Mediolateral (ML)* Frequency with 95% of signal (F95) *Anteroposterior (AP) Mediolateral (ML)* Mean sway velocity *Anteroposterior (AP) Mediolateral (ML)*	Quiet Stance	For RMS accelerations, a significant main effect for group showed that PD participants had greater ML accelerations than controls, while the AP axis fell marginally short of statistical significance. PD participants also had higher AP and ML Jerk scores at baseline, but ML Jerk was also larger for the PD patients at the 3–6 and 12-month follow-up time points. There were also significant main effects for group for ML F95 values and mean sway velocity along the ML axis, indicating that the PD group had larger values for both of these measures compared with control.
Mirelman 2013 ^[^ ^36^ ^]^	***PD LRRK2 Gene*:** Carrier = 50 (62.6±9.6) Non-Carrier = 50 (60.2±11.3)	***Hoehn & Yahr*** Carrier = 2–3 Non-Carrier = 2–3 ***UPDRS Total*** Carrier = 27.9±14.2 Non-Carrier = 26.9±13.3	Carrier 4.4±3.3 Non-Carrier 6.1±6.1	3D Accelerometer Freq: Not reported Lower back	***Preferred vs*. *Fast speed vs*. *Dual-task*:** Stride timing variability Step regularity (step-to-step consistency) Width of dominant frequency *Anteroposterior (AP)*	Gait	Carriers of the LRRK2 gene had greater stride timing variability and less step regularity than non-carriers during preferred speed, fast speed and dual-task (serially subtracting 3s) walking. Carriers also had greater gait variability during preferred and fast walking, as evidenced by the greater width of the dominant frequency. Significant group by condition interactions suggested that the carriers had a greater increase in stride timing variability and a greater width of the dominant frequency with increased task complexity (i.e. dual tasking) compared with non-carriers.
Palmerini 2013 ^[^ ^14^ ^]^	PD = 20 (62.0±7.0) Control = 20 (64.0±6.0)	***Hoehn & Yahr*** PD = 2.4±0.2	PD 5.2±4.1	3D Accelerometer Freq: 100 Hz L5	RMS acceleration *Anteroposterior (AP) Mediolateral (ML) Vertical (VT)* Normalised Jerk *Anteroposterior (AP) Mediolateral (ML) Vertical (VT)* Harmonic ratio (HR) *Anteroposterior (AP) Mediolateral (ML) Vertical (VT)* Phase coordination index	Timed Up and Go	During the gait and turning portions of the Timed Up and Go test, PD patients had significantly lower AP and ML normalised Jerk scores than control participants. Similarly, during the gait component of the test, PD participants also had lower AP and VT HRs compared with controls. The two groups did not differ significantly for any of the other accelerometer-based measures.
Palmerini 2011 ^[^ ^40^ ^]^	PD = 20 (62.0±7.0) Control = 20 (64.0±6.0)	***Hoehn & Yahr*** PD = ≤2.5 ***UPDRS-III*** PD = 26.6±7.1	Not Reported	3D Accelerometer Freq: 100 Hz L5	High Frequency Power *Anteroposterior (AP) Mediolateral (ML)* Frequency Dispersion *Anteroposterior (AP) Mediolateral (ML)* Sway Range *Anteroposterior (AP) Mediolateral (ML)*	Quiet Stance	Compared with controls, the PD group had significantly higher high frequency power in the ML direction during the dual task condition and significantly lower AP frequency dispersion scores while standing on a foam surface. AP sway range was not significantly different between groups. A wrapper feature selection approach determined that ML high frequency power on a firm surface with eyes open, AP frequency dispersion on a foam surface with eyes open and AP sway range on foam surface with eyes closed represented the best candidate subset to distinguish PD from controls.
Rocchi 2014 ^[^ ^33^ ^]^	PD PIGD = 40 (64.5±6.9) PD TD = 26 (67.6±9.9) Control = 15 (78.2±3.9)	***UPDRS III*** PD PIGD = 38.3±10.9 PD TD = 43.3±13.4	PD PIGD 5.1±3.6 PD TD 5.7±2.8	3D Accelerometer Freq: 100 Hz Lower back	***Feet together vs*. *Semi-tandem*:** Centroidal frequency (CF) *Anteroposterior (AP)* Length of sway *Anteroposterior (AP) Mediolateral (ML) 2-dimensional (2D)* Mean sway velocity *Anteroposterior (AP) Mediolateral (ML)*	Quiet Stance	The TD group had significantly lower CF values than controls for all experimental tasks and the PIGD group also had lower CF values than controls for all conditions except semi-tandem stance with eyes closed. The TD and PIGD groups did not differ with respect to CF during any of the experimental tasks. CF values were influenced by foot position for the two PD groups (PIGD and TD) with greater values recorded during semi-tandem stance. Results were similar for sway velocity and length of sway, with all groups typically showing higher values with eyes closed compared with eyes open. The groups did not differ for sway velocity or length of sway for the feet together or semi-tandem stance trials with eyes open, but the PIGD and TD groups had lower values compared with controls during the EC conditions.
Sant’Anna 2011 ^[^ ^27^ ^]^	PD = 11 (60.0±8.6) Control = 11 (61.0±7.8)	***Hoehn & Yahr*** PD = 1.6±0.6 ***UPDRS-PIGD*** PD = 0.7±1.1	PD 1.1±1.1	1D Gyroscopes Freq: 200 Hz Anterior shank 2D Gyroscopes Freq: 200 Hz Wrist	Symbolic symmetry index (SI_symb_) Symmetry index (SI_index_) Gait asymmetry (SI_GA_) Symmetry angle (SI_angle_) Maximum angular velocity ratio (SI_ratio_) Trend symmetry (SI_trend_) LCEA symmetry magnitude (SI_LCEA_)	Gait	Of the symmetry measures derived from the gyroscopes placed on the shanks and wrists, only the SI_index_, SI_GA_, SI_ratio_ and SI_symb_ values for the wrist sensors were significantly higher for PD participants. Evaluation of the area under the Receiver Operating Characteristic (ROC) curves for these four outcomes showed that only SI_ratio_ and SI_symb_ were able to differentiate PD from controls, but the higher Intra-class Correlation Coefficients for SI_symb_ indicated that this outcome was more robust for differentiating between the two cohorts.
Sejdić 2014 ^[^ ^20^ ^]^	PD = 10 (≥65 years) Neuropathy = 11 (≥65 years) Control = 14 (≥65 years)	***Hoehn & Yahr*** PD = 2–3	Not Reported	3D Accelerometer Freq: 100 Hz L3	Lyapunov exponent (LE) *Anteroposterior (AP) Mediolateral (ML) Vertical (VT)* Harmonic ratio (HR) *Anteroposterior (AP) Mediolateral (ML) Vertical (VT)* Entropy rate *Anteroposterior (AP) Mediolateral (ML) Vertical (VT)* Cross entropy rate *Anteroposterior (AP) Mediolateral (ML) Vertical (VT)*	Gait	There were no significant differences between the groups for AP, ML or VT Lyapunov exponents, but PD patients had less gait rhythmicity in the vertical direction (decreased VT HRs) compared with healthy controls. With respect to the entropy measure, the PD and peripheral neuropathy groups both had significantly greater ML values than controls, but there were no group differences for cross entropy rate.
Sekine 2004 ^[^ ^21^ ^]^	PD = 11 (66±9.6) Control = 10 (66.3±5.3)	***Hoehn & Yahr*** PD = 1–2	Not Reported	3D Accelerometer Freq: 1024 Hz L5/S1 region	Fractal Brownian Motion *Anteroposterior (AP) Mediolateral (ML) Vertical (VT)*	Gait	The fractal values for the AP, ML and VT directions were significantly higher for the individuals with PD compared with controls. Also, the AP, ML and VT fractal dimensions were all significantly negatively correlated with walking speed for the PD group, but not controls.
Sekine 2004 ^[^ ^34^ ^]^	Mild PD = 11 (66.0±9.6) Severe PD = 5 (57.4±19.1) Control = 10 (66.3±5.3)	***Hoehn & Yahr*** Mild PD = 1–2 Severe PD = 3–4	Not Reported	3D Accelerometer Freq: 1024 Hz L5/S1 region	Vertical patterns *Anteroposterior (AP) Mediolateral (ML) Vertical (VT)* Circular patterns *Anteroposterior (AP) Mediolateral (ML) Vertical (VT)* Horizontal patterns *Anteroposterior (AP) Mediolateral (ML) Vertical (VT)*	Gait	Controls did not differ significantly from the mild or severe PD groups for AP, ML or VT vertical patterns. Circular patterns were different between the groups, with both mild and severe PD participants having larger values than controls in the AP and VT directions, while severe PD patients also had higher AP circular patterns than mild PD patients. Severe PD patients had greater short horizontal patterns than controls in all three directions and lower long horizontal patterns in the AP and VT than controls. Severe PD patients also had greater short horizontal patterns in the AP, ML, VT than mild PD patients and mild PD patients had lower values than controls for long horizontal patterns in the AP and VT directions.
van Emmerik 1999 ^[^ ^29^ ^]^	PD = 27 (53.7±10.6) Control = 11 (*not reported*)	***Hoehn & Yahr*** PD = 1.5 ±0.6 ***UPDRS III*** PD = 16.7±6.2	PD 2.3±1.4	1D Accelerometer Freq: 104 Hz Shank	Stride timing variability Relative phase analysis	Gait	Stride timing variability was not significantly different between PD and controls, but variability significantly decreased for both groups as walking velocity increased. Continuous relative phase was also larger for controls compared with PD patients between walking speeds of 0.2 and 1.4 m/s.
Weiss 2011 ^[^ ^22^ ^]^	PD = 22 (65.9±5.9) Control = 17 (69.9±8.8)	***Hoehn & Yahr*** PD = 2.5±0.4 ***UPDRS III*** PD = 23.6±9.4	PD 4.8±3.8	3D Accelerometer Freq: 256 Hz Lower back	Stride timing variability Width of the dominant harmonic	Gait	Stride timing variability was significantly higher for PD patients compared with healthy controls. Similarly, the width of the dominant harmonic of the power spectral density of the locomotor band of the acceleration signal was significantly greater for PD patients, both on and off medication, compared with controls. Furthermore, the width of the dominant harmonic was greater for patients when off medication compared with on medication.
Weiss 2014 ^[^ ^35^ ^]^	***PD Freezer vs*. *Non-Freezer*:** Non-Freezer = 44 (66.5±8.8) Freezer = 28 (64.4±8.7)	***Hoehn & Yahr*** Non-Freezer = 2.4±0.5 Freezer = 3.2±0.8 ***UPDRS III—OFF*** Non-Freezer = 42.3±12.9 Freezer = 46.2±12.2 ***UPDRS III—ON*** Non-Freezer = 35.6±12.8 Freezer = 36.3±11.7	Non-Freezer 6.7±2.2 Freezer 7.5±4.5	3D Accelerometer Freq: Not reported Lower back	Harmonic ratio (HR) *Anteroposterior (AP) Mediolateral (ML) Vertical (VT)* Stride regularity *Anteroposterior (AP) Mediolateral (ML) Vertical (VT)* Width of dominant frequency *Anteroposterior (AP) Mediolateral (ML) Vertical (VT)*	Gait	Freezers had decreased AP, ML and VT harmonic ratios and stride regularity compared with non-freezers. PD freezers also had a significantly greater width of the dominant frequency in the VT and AP directions. Harmonic ratios and stride regularity were significantly correlated with the new freezing of gait questionnaire (NFOG-Q) and the width of the dominant frequency in the VT and AP direction were also significantly correlated with this clinical test.
Weiss 2014 ^[^ ^31^ ^]^	***PD Fallers vs*. *Non-Fallers*:** Non-Faller = 67 (64.0±9.8) Faller = 40 (66.5±8.2)	***Hoehn & Yahr*** Non-Faller = 2.4±0.5 Faller = 2.9±0.8	Non-Faller 5.2±3.1 Faller 6.1±4.0	3D Accelerometer Freq: 100 Hz Lower back	Harmonic ratio (HR) *Anteroposterior (AP) Mediolateral (ML) Vertical (VT)* Stride regularity *Anteroposterior (AP) Mediolateral (ML) Vertical (VT)* Width of dominant frequency *Anteroposterior (AP) Mediolateral (ML) Vertical (VT)*	Gait	During a 3-day assessment of gait and mobility, fallers exhibited reduced HRs in both the AP and VT directions. PD fallers also had less VT stride regularity than non-fallers and a greater width of the dominant frequency for the AP and VT directions.
Yang 2011 ^[^ ^23^ ^]^	PD = 5 (78.0±9.8) Control = 5 (26.0±3.1)	***Hoehn & Yahr*** PD = 2–3	Not Reported	3D Accelerometer Freq: 50 Hz Lateral pelvis	Step regularity Stride regularity Step symmetry	Gait	There were no significant differences observed in step regularity, stride regularity or step symmetry between PD patients and controls.
Zampieri 2009 ^[^ ^28^ ^]^	PD = 12 (60.4±8.5) Control = 12 (60.2±8.2)	***Hoehn & Yahr*** PD = 1.6±0.5 ***UPDRS III*** PD = 20.0±9.4	PD 1.1±1.1	1D Gyroscopes Freq: 200 Hz Anterior shank 2D Gyroscopes Freq: 200 Hz Wrist Inertial Sensor Freq: 200 Hz Sternum	Stride length variability Stride timing variability	Timed Up and Go	PD and control groups did not differ with respect to stride length variability or stride time variability.

**PD:** Parkinson’s disease; **PSP:** Progressive supranuclear palsy; **IPD:** Idiopathic Parkinson’s disease; **VPD:** Vascular Parkinson’s disease; **HRPD:** People at high-risk of Parkinson’s disease; **UPDRS:** Unified Parkinson’s Disease Rating Scale; **MDS-UPDRS:** Movement Disorders Society’s revision of the Unified Parkinson’s Disease Rating Scale; **Freq:** Sampling frequency of wearable sensor

In addition to extracting and compiling these data, a quality assessment was performed by using a modified version of a previously-developed 27-item quality checklist, designed to accommodate both randomised and non-randomised studies [14]. To evaluate the overall methodological quality of each paper, 25 of the criteria on the quality assessment tool were assigned a score of one point if the criterion was met or a zero if the criterion was not met. If it was not possible or unreasonably difficult for the assessors to determine whether the information required for a particular criterion had been provided by the authors, a score of zero was given for that criterion. Of the remaining two questions on the quality checklist, one question evaluating whether potentially confounding variables had been reported by the authors was assessed on a 2-point scale, where the study was given 2 points if confounders were clearly described, 1 point if they were partially described or 0 points if they were not described. The final methodological aspect of the studies that was evaluated was statistical power, which was more heavily weighted than the other criteria and assessed on a 5-point scale. Studies that achieved a statistical power of ≤70% for the standing balance or walking stability measures were given a score of zero, while those that achieved powers of 80, 85, 90, 95 or 99% were assigned scores of 1 to 5, respectively. Where an appropriate statistical power calculation was not provided by the authors, it was necessary to evaluate the statistical power of each study based on the data presented by the authors. If a statistical power calculation was not reported and the raw data were not presented, the paper was given a score of zero for this criterion. After each paper was assessed against these criteria, the scores were summed and divided by the maximum total points to yield a final score that represented the percentage of total possible points earned. This percentage score was used to evaluate the overall quality of the study using quartiles to classify the methodological quality of the article as either very low (≤25%), low (>25%, but ≤50%), moderate (>50%, but ≤75%) or high (>75%). The methodological quality assessment tool (S2 File) and the scoring of each of the studies included in this review (Table A in S2 File) are provided as Supporting Information.

## Results

The initial database search identified 335 articles that were potentially eligible for inclusion in this review. Of the 335 studies identified, 98 were excluded as duplicates, 114 were conference abstracts, six were review articles and six were written in a language other than English. The remaining 115 papers were screened by title and abstract, which resulted in 34 being excluded, based on title and 38 being excluded based on abstract. A manual search was conducted of the bibliographies of those papers that were considered appropriate for full-text review, which identified five additional papers for consideration. Following full-text review of the remaining 44 studies, a further 18 studies were excluded, including one that was unattainable, three that had no control group or condition and 14 that had no sensor-based measure of standing balance or walking stability. The remaining 26 articles were selected for inclusion in this systematic review.

### Study Design and Methodological Quality

All 26 studies included within this review had a cross-sectional research design with a broad aim of using different types of wearable sensors to observe or identify differences in standing balance or walking stability for Parkinson’s disease compared with controls or a control condition (e.g. on medication vs. off medication, PD subtypes). Given their cross-sectional nature, ten items were excluded from the methodological quality checklist, as they specifically targeted qualities that are unique to intervention studies. The decision to exclude these criteria was made to ensure that the overall quality of the studies included in this review was not unfairly biased by these items that were not relevant to their chosen design.

Based on the appraisal of methodology quality, eight papers were identified as being of low methodological quality (range = 31.8% to 50.0%), 15 papers were of moderate methodological quality (range = 54.5% to 72.7%) and three papers were of high methodological quality (range = 77.3% to 90.9%). In general, the reviewed papers performed poorly on criteria addressing external validity (e.g. representativeness of the sample), internal validity (e.g. identification of and adjustment for potential confounders) and statistical power (e.g. no power calculation and insufficient details to make an informed appraisal).

### Sensor Type and Placement

Multiple wearable sensor types were used within the included articles to assess measures of standing balance and walking stability. Of these studies, 69% reported using three-dimensional accelerometers [14, 17–23, 30–37, 39, 40], 27% used inertial sensors [13, 24–28, 38], and 4% used other types of sensors [28, 29]. Similarly, there were multiple protocols described with respect to the placement of the wearable sensors on the human body. Of the 26 included studies, 85% reported placing a wearable sensor on either the lumbar or sacral region of the trunk [13, 14, 17–22, 24–27, 31–40] and 15% reported placing devices on other body landmarks (e.g. head, shank, wrist) [23, 28–30]. Details on the studies included in this review that reported using each specific type and placement of sensors are summarised in Table 1.

### Assessment of standing balance and walking stability

Of the 26 included studies, 65% used wearable sensors to assess walking during clinical tests, such as the Timed up and Go Test [14, 28] or during assessments of straight-line walking at a self-selected speed [17–23, 27, 29–31, 34–36, 39]. A wide range of sampling frequencies was used to assess walking stability in the reviewed studies, with authors reporting sampling frequencies ranging between 20 and 1024 Hz. The remaining nine studies (35%) assessed standing balance using an instrumented functional reach test [37], dynamic posturography [24] or one of many pre-existing clinical tests conducted during quiet stance (i.e. the Romberg test, tandem stance, semi-tandem stance, standing with eyes open and eyes closed) [13, 25, 26, 32, 33, 38, 40]. Understandably, the wearable sensors used in these studies were generally set to collect data at a slower rate to those used for assessing the dynamic tasks, with reported sampling frequencies ranging from 50 to 128 Hz.

The included studies reported multiple outcomes of standing balance and walking stability that were calculated from the signals provided by the wearable sensors (e.g. accelerations). Of these outcomes, the most commonly-reported measures of standing balance included postural sway velocity (23% of studies) [13, 25, 26, 32, 33, 38], RMS accelerations (19% of studies) [13, 24–26, 38] and jerk (19% of studies) [13, 25, 26, 37, 38]. The most commonly-reported measures of walking stability included, the harmonic ratio (31% of studies) [14, 17, 19, 20, 22, 30, 35, 39] and stride timing variability (27% of studies) [17, 19, 22, 28–30, 36]. A summary of the studies reporting each of the outcome measures of standing balance and walking stability is provided in Table 2.

**Table 2:** Summarises and defines the sensor-based measures of standing balance and walking stability used in the studies included in this review.

Outcome Measure	Definition of Measure	Articles
**Standing Balance or Walking Stability**		
*Mean acceleration*	The average of the anteroposterior (AP), mediolateral (ML) or vertical (VT) accelerations during a specific phase of the movement. Provides an indication of the rate of change in the velocity of the body during this phase. Under static conditions, larger values would represent poorer control.	[37]
*Root mean square (RMS) acceleration*	Taking the RMS of the accelerations makes all values of the time series positive, to yield an average positive amplitude for AP, ML or VT accelerations. Like mean accelerations, RMS accelerations provides an indication of the rate of change in velocity, but is more robust for data that has both positive and negative values.	[13,14,18,24–26,30,38]
*Jerk*	Time series of the first derivative of acceleration (third derivative of displacement), representing the rate of change of acceleration. It is calculated from the raw AP, ML or VT accelerations. During steady movements, the body should be neither accelerating nor decelerating rapidly, hence Jerk scores should be smaller for more stable people.	[13,25,26,37,38]
*Root mean square (RMS) Jerk*	Similar to RMS accelerations, RMS Jerk mathematically converts all values to a positive number and provides an average value for the AP, ML and VT Jerk time series. In lay terms, the RMS Jerk provides a single value that describes the jerkiness of the movement.	[18]
*Normalised Jerk*	RMS Jerk score divided by overall movement time. Provides similar information to RMS Jerk, but takes into account differences in task duration for different populations.	[14]
**Standing Balance**		
*Maximum sway distance*	The resultant of AP and ML displacement is calculated for an inertial measurement unit placed at the height of the centre of mass (COM; 55% of height). Maximum sway distance is the single largest value recorded throughout the trial. Provides insight into the extremes of postural sway.	[32]
*Mean sway distance*	The resultant of AP and ML displacement is calculated for an inertial measurement unit placed at the height of the COM (55% of height). Mean sway distance is the average of all resultant values recorded throughout the trial. Larger values represent poorer postural control.	[13,32]
*Sway Range*	The overall range of displacement of the centre of mass (COM; estimated from an inertial measurement unit positioned on the trunk) in the anteroposterior (AP) and mediolateral (ML) directions. Larger values represent an increased amount of postural sway.	[40]
*Length of sway*	The total distance travelled by the COM on the transverse plane. Increased length of sway indicates more sway per unit of time and, hence, reduced postural control.	[13,32,33]
*Mean sway velocity*	The first integral of the AP, ML or VT acceleration signals. Higher sway velocities represent more erratic postural adjustments and, hence, poorer postural control.	[13,25,26,33,38]
*Sway area*	The elliptical area that encapsulates the sway path derived from the AP and ML accelerations. Larger sway areas represent an increased volume of sway, which may suggest poorer balance.	[13]
*F95*	The frequency below which 95% of the acceleration signals power is present. Higher frequencies would represent a larger number of postural adjustments to maintain balance during the trial.	[13,25,26,38]
*Centroidal frequency*	The frequency at which the power of the signal above and below are exactly balanced (i.e. the centre point). The centroidal frequency can be calculated for the AP, ML and VT axes separately. Lower frequencies represent poorer postural control.	[33]
*High frequency power*	Percentage of the acceleration signal that is present between 4 and 7 Hz. A greater proportion of data in this high frequency band represents increased postural adjustment and postural sway.	[40]
*Frequency dispersion*	A unitless frequency-based measure of variability. Values closer to zero would represent more regular patterns of sway, while values closer 1 represent a greater degree of variability.	[40]
**Walking Stability**		
*Harmonic Ratio*	A measure of the stability of gait-related accelerations by evaluating the stride-to-stride regularity of the harmonics within the acceleration signal. Walking patterns that produce higher ratios have more regular acceleration profiles over successive gait cycles (i.e. less stride-to-stride variability); hence, the gait pattern is deemed to be more stable.	[14,17,19,20,30,31, 35,39]
*Step and stride regularity*	The regularity of the AP, ML or VT acceleration profiles from step-to-step or stride-to-stride. Higher regularity scores represent a more rhythmic and consistent walking pattern and is often said to reflect a more stable gait pattern.	[17,23,31,35,36]
*Step symmetry*	Ratio of step regularity to stride regularity. A ratio closer to 1 represents greater symmetry between the left and right steps, while values closer to 0 indicate poorer symmetry.	[23]
*Step and stride timing variability*	The standard deviation (SD) or the coefficient of variation ((SD/mean)*100) of all step or stride times collected during a trial. Greater variability represents a less rhythmic walking pattern that is often said o reflect a less stable gait pattern.	[17,19,22,28–30,36]
*Stride length variability*	The standard deviation (SD) or the coefficient of variation ((SD/mean)*100) of all stride lengths collected for the left and right leg collected throughout a trial. Greater variability represents a less predictable and, hence, less stable walking pattern.	[19]
*Lyapunov exponent*	A non-linear measure that assesses the sensitivity of the system to perturbations in the AP, ML or VT directions. The Lyapunov exponent provides an indication of the local dynamic stability of the gait pattern, with lower values representing increased local stability during gait.	[20]
*Entropy rate*	Assesses the regularity of the AP, ML and VT accelerations. Values range from 0, which represents no regularity (maximum randomness) to 1, which represents maximum regularity.	[20]
*Cross entropy rate*	Non-linear measure of asynchrony between two related time series. Used to assess how well the pattern of AP acceleration (for example) can predict ML accelerations. Higher values indicate more synchronisation between the acceleration patterns and, hence, a more stable gait pattern.	[20]
*Width of the dominant frequency*	The width of the dominant harmonic of the power spectral density of the acceleration signal. Greater widths, represent greater dispersion and greater variability of the gait pattern.	[22,31,35,36]
*Relative phase analysis*	A graphic-based analysis that plots the angular position of a segment against the angular velocity of the same segment. Relative phase analysis provides a measure of the coordination between two adjoining segments (e.g. pelvic and trunk) and the overall stability of this pattern.	[29]
*Phase coordination index (PCI)*	Stable walking has step times that are approximately half the length of the gait cycle (i.e. 180° of a 360° cycle). Deviation from this expectation is considered an inaccuracy. The PCI is a summary measure that combines this value representing the accuracy with the coefficient of variation, representing consistency, hence the PCI is considered a measure of gait coordination.	[14]
*Symmetry index (SI*_*index*_ *)*	The SI_index_ compares movements from one side (e.g. injured) to the other side (e.g. uninjured). Perfect symmetry is represented by zero and larger numbers represent more asymmetry.	[27]
*Gait asymmetry (SI*_*GA*_ *)*	Mean swing time is calculated for both left and right legs. Gait asymmetry is the natural log (ln) of the swing time of the leg with the shortest swing time divided by the swing time of the leg with the longer swing time. Values closer to zero represent a symmetrical movement pattern.	[27]
*Symmetry angle (SI*_*angle*_ *)*	Measures the relationship between discrete values obtained from the left and right side and is derived when the right-side value is plotted against the left-side value to create a line that forms an angle with the x-axis. Angles that deviate from 45° represent some degree of asymmetry.	[27]
*Maximum angular velocity ratio (SI*_*ratio*_ *)*	Ratio of the maximum angular velocity of the left leg (averaged over all gait cycles) to maximum angular velocity of the right leg (averaged over all gait cycles). Values that are closer to zero represent better symmetry between the left and right sides of the body.	[27]
*Trend symmetry (SI*_*trend*_ *)*	Translated data from the left and right sides of the body are used to derive eigenvectors. Trend symmetry assesses the ratio of the variability *about* the eigenvector (y-axis) to the variability *along* the eigenvector (x-axis). A value of zero represents perfect symmetry.	[27]
*LCEA symmetry magnitude (SI*_*LCEA*_ *)*	Applies a latency corrected ensemble average (LCEA) to assess the correlation between the magnitudes of the signals collected from the left and right sides of the body using a cross-correlation approach. Larger values represent a greater degree of symmetry.	[27]
*Fractal Brownian Motion*	Fractal measures provide an indication of the complexity of the AP, ML, VT accelerations during walking. Higher values represent more complex walking patterns, hence walking patterns that are more difficult to coordinate and control effectively.	[21]
*Vertical Patterns*	A time-frequency pattern of the energy of the acceleration signal for AP, ML and VT directions. Vertical patterns represent impulse type activities during the walking cycle.	[34]
*Circular Patterns*	A time-frequency pattern of the energy of the acceleration signal for AP, ML and VT directions. Circular patterns characterise irregular burst like patterns during the walking cycle.	[34]
*Horizontal Patterns*	A time-frequency pattern of the energy of the acceleration signal for AP, ML and VT directions. Horizontal patterns represent long-term smooth and regular activities.	[34]

## Discussion

The purpose of this systematic review was to examine the existing literature to determine the best types of wearable sensors and the most appropriate anatomical placements and outcome measures to assess deficits in balance and gait between people with PD and controls. Using the methodological quality assessment tool adapted from Downs and Black [41], it was determined that the overall quality of scientific reporting in this area is largely of low to moderate quality. In general, the reviewed papers were lacking details concerning the representativeness of the study population (external validity), the approaches adopted to identify and account for confounding variables (internal validity) and an appropriate justification for the chosen sample size. Interestingly, 62% of the included studies received a score of zero for all of the criteria related to at least two of these three areas, while one study (4%) received a score of zero for all three of these areas. The heavier weighting attributed to the sample size criterion is indicative of the importance of ensuring that a study has sufficient statistical power to identify a difference where one exists and, hence, minimise the likelihood of incorrectly accepting the null hypothesis (i.e. Type II error) [42]. Of the 26 studies included in this review, not one reported the results of a sample size calculation, but 13 (50%) had fewer than 15 participants in each of their groups [13, 19–21, 23–28, 32, 34, 39] and three others (12%) had at least one group with fewer than this number [29, 37, 38]. While it is important to emphasise that a large sample size is not always required to address a specific research question, reporting the outcome of an appropriate a-priori statistical power calculation is beneficial for determining the overall rigor of the reported findings.

Of the other methodological aspects that were poorly reported, the lack of appropriate detail regarding the influence of confounding variables was quite substantial, as failure to account for these factors may result in a study observing a significant change that is simply the manifestation of another variable not adequately controlled for [43]. For example, it is widely recognised that gait and balance variables are influenced by walking speed [44–48] and age [49–51], hence if groups differ for either or both of these variables, appropriate adjustments should be made to account for this. Of the reviewed studies, 15 (58%) described the principal confounder(s) of their research and reported having made adjustments to their outcomes to account for these variable(s) [17, 19, 22, 26–32, 35, 36, 38–40]. Of the remaining studies, four (15%) provided a description of the potential confounders, but lacked clear descriptions of how they were accounted for in their analyses [14, 21, 34, 37], while seven (27%) neither reported nor accounted for their potential confounders [13, 18, 20, 23–25, 33]. In the study by Fazio et al [18], it was reported that people with PD had significantly lower accelerations and jerk scores than ataxic patients and healthy controls. However, the age of the patients in the PD group (n = 17) ranged from 60–85 years, while the ataxic patients (n = 24) and controls (n = 24) were aged between 20 and 85 years, with more than 60% of these participants aged less than 60 years. Furthermore, the authors reported that the PD and ataxic patients walked significantly slower than the control participants. Given the differences in age and walking speed between the cohorts, it is difficult to determine whether the reported differences in acceleration profiles were indicative of disease-related changes or whether they were simply representative of age-related and/or speed-related factors. Identifying all potential confounders in this type of research and reporting how they have been accounted for in the analyses is critical to ensuring that any changes in outcome can be confidently attributed to the treatment or disease of interest. Collectively, the results of the methodological quality assessment identified that issues related to internal and external validity, as well as statistical power are typically poorly reported in the literature. It should be emphasised that this does not suggest that the authors did not consider some or all of these factors, but rather suggests that these areas should be given more attention in the reporting of future research. To improve the overall methodological quality of research in this area, it is recommended that scientists use existing research reporting guidelines (e.g. CONSORT, STROBE) when designing and planning the reporting of their studies.

Despite the outlined shortcomings in the reporting of the methods, 81% of the studies described differences between different PD groups and/or a healthy control group for one or more of their sensor-based measures of standing balance or walking stability [13, 14, 17–22, 25–27, 29–37, 39, 40]. However, contradictory findings reported in separate studies suggest that some of the reported outcomes may be more robust than others. For example, two studies that compared PD patients with controls using a standing balance assessment reported no significant differences between the groups for jerk scores [37, 38], while three others reported significantly greater jerk scores for PD patients [13, 25, 26]. Similarly, two studies reported no differences between people with PD and controls for RMS accelerations [24, 38], while three studies reported significantly greater RMS accelerations for PD patients [13, 25, 26]. Sway velocity was another common measure used to evaluate standing balance, but similarly only three studies [25, 26, 33] reported differences between people with PD and controls, while the remaining three did not [13, 32, 38]. It is interesting to note, however, that contradictory findings were presented by the three studies reporting differences between patients and controls for sway velocity, as one study reported reduced values for PD patients while standing with eyes closed [33], while the others reported greater values for people with PD while standing with eyes open [25, 26], but not eyes closed [26]. While each of the studies that assessed standing balance derived their outcomes from a wearable sensor positioned on the trunk [13, 24–26, 32, 33, 37, 38], there were some methodological differences that may explain the discrepancies observed between the studies’ reported outcomes. The studies unable to report significant differences in jerk scores, RMS accelerations and sway velocities assessed standing balance using a semi-tandem stance test [38], the Sensory Organisation Test [24], the Romberg test [32] or an instrumented version of the functional reach test [37]. In contrast, the studies that reported significant differences for jerk, RMS accelerations and sway velocities assessed participants during quiet standing with the heels separated by 10 cm [13, 25, 26] or while they stood with their feet together or in a semi-tandem stance with their eyes open and closed [33]. Given the available evidence, it seems that the best recommendation for clinicians seeking to assess standing balance using wearable sensors would be to calculate RMS accelerations or jerk scores from trunk accelerations collected while patients stand with their eyes open and their heels 10 cm apart. However, a degree of caution may be required when considering this recommendation, as three of the four studies that reported differences in standing balance for people with PD appear to have used the same patient cohort, due to the reported demographics being the same for each study [13, 25, 26]. As such, it is possible that the overall interpretation of the existing literature in this area may be biased and the transferability of the findings may be more limited than they appear.

In addition to the nine studies that used wearable sensors to assess standing balance, the remaining 65% used these devices to assess walking stability. These studies reported numerous outcome measures derived from the acceleration signals, but the Harmonic Ratio (HR) was the most commonly-reported measure and was calculated for the head [30] and lumbosacral region [14, 17, 19, 20, 30, 31, 35, 39]. The HR seems to be a sensitive and versatile measure of walking stability, as the reviewed literature reports differences between people with PD and controls [14, 19, 20, 30], PD freezers and non-freezers [35], PD fallers and non-fallers [30, 31], PD patients with different dominant symptoms [17] and different methods of cueing for people with PD [39]. Stride timing variability was the second most common outcome measure for the studies that assessed walking stability, but careful review of the included studies suggested that it may not be a dependable measure for discriminating between different populations. Of the seven studies that reported this outcome, three described differences in stride timing variability between PD fallers and non-fallers [30], PD patients and controls [22, 30] or carriers and non-carriers of the LRRK2 gene mutation [36]. In contrast, four studies reported no differences between PD patients and controls [19, 28, 29] or patients with different sub-types of PD [17]. A common characteristic of those studies reporting differences for the HR and stride timing variability was that they each assessed walking stability during straight line walking. As such, it is recommended that clinicians who wish to assess walking stability using wearable sensors calculate the HR from trunk accelerations collected while patients walk in a straight line at a self-selected speed. While there is some evidence to support the use of stride timing variability to assess walking stability, it would only be recommended as a secondary measure due to the inconsistencies evident within the current literature.

While it was not the primary focus of this review to evaluate the effects of anti-parkinsonian medications, such as levodopa, on measures of standing balance and walking stability, it is an important factor that warrants consideration. It is widely recognised that levodopa improves symptoms of PD (based on the UPDRS) [17, 32], spatiotemporal gait characteristics (e.g. stride length) [52, 53] and performance on clinical tests of balance, such as the Berg Balance scale [54]. Of the studies included in this review, five (19%) reported assessing standing balance or walking stability while patients were not medicated [14, 24, 33, 38, 40], 9 (35%) assessed patients on-medication [18–21, 30, 31, 35, 36, 39] and three (12%) assessed patients in both on and off states [17, 22, 32]. Of the remaining studies, six (22%) assessed patients who were not yet being medicated for PD [13, 25–29], while three (12%) did not report whether their participants were on or off medication at the time of testing [23, 34, 37]. Interestingly, of the studies not reporting differences in standing balance or walking stability between different groups of PD patients and/or healthy controls, two assessed patients while they were off medication [24, 38], while the other did not report whether patients were assessed on or off medication [23]. Of the three studies that assessed patients on and off medication, only two statistically compared their presented outcomes for the two conditions [22, 32]. For a group of idiopathic PD patients, it was reported that the length and maximal distance of postural sway was significantly increased during normal stance, when patients were assessed on medication [32], which would typically be interpreted as a greater amount of sway during the medicated state. During walking, Weiss et al. [22] reported a significant reduction in the width of the dominant harmonic in the acceleration signal when patients were tested on medication, which represented less variability in the gait patterns of medicated patients. While there is a clear need for further research in this area, the presented findings suggest that wearable sensors can be effectively used to evaluate changes in standing balance and walking stability for different patients who are assessed with or without anti-parkinsonian medication.

Considering that 66% of individuals with PD fall at least once in a given year [11, 55] and nearly 50% of these falls occur during locomotion [56, 57], assessing walking stability and falls risk is critical to ensure that high-risk patients can be easily identified by clinicians. However, to date, there is a paucity of research evaluating the capacity for wearable sensors to identify people with PD who are at a higher risk of prospectively falling. Two of the studies included in this review compared people with PD who retrospectively reported having no falls (non-fallers) to those who reported falling at least once (fallers) in the previous 12 months [30, 31]. Both of these studies reported that PD fallers had less rhythmic movements for the pelvis or lower trunk (as assessed using the HR) in both the anterior-posterior (forward-backward) and vertical directions compared with PD non-fallers [30, 31] and controls [30]. While their retrospective nature makes it difficult to determine whether these deficits contribute to the patients falling or whether they are perhaps a consequence of an increased fear of future falls, the results of these studies provide some support for the use of wearable sensors for screening patients for falls risk. Nevertheless, further prospective research is needed to confirm whether sensor-based measures of standing balance or walking stability are suitable for the assessing falls risk and predicting future falls in this population.

There are a number of limitations that should be considered when interpreting the results of this review of literature. First, the results of the methodological quality assessment included in this systematic review are based on the assessor’s (RPH) interpretation of each of the studies. Often, the results reflect the quality of the reporting of the research and, hence, should not be seen as a critique of the significance of the research and its outcomes. Second, given the relatively small number of studies published in this area and the wide variety of research questions addressed using wearable sensors, it is difficult to make strong recommendations regarding the most appropriate equipment, placements and outcomes for assessing standing balance and walking stability in people with PD. In light of these limitations, the results presented in this systematic review should be considered preliminary and additional work will be required as this field of science continues to evolve.

In conclusion, wearable sensors provide a light-weight, portable and affordable alternative to more expensive three-dimensional motion analysis systems and are effective for detecting changes in standing balance and walking stability among people with PD. However, it appears that some outcome measures may be more useful than others for discriminating patient cohorts from controls. Specifically, measures of jerk and RMS acceleration for the trunk appear to be the best sensor-based measures of standing balance, even under less challenging conditions (i.e. feet apart on a firm surface with eyes open). For assessments of walking stability, a trunk-mounted wearable sensor can be used to assess the rhythmicity of dynamic gait patterns using the HR calculated for the three axes of motion. While some studies have provided support for other more complex frequency-based measures of postural stability, additional research is essential to objectively assess the utility of these measures for the PD population. Future research should give careful consideration to the internal and external validity of their methods and provide an appropriate sample size calculation to support their study, as these aspects could have been better reported in the existing literature.

## Supporting Information

S1 File Systematic search strategy and procedures. (DOCX)

S2 File The quality of methodological reporting assessment tool and the outcomes of this assessment for each of the included studies. (DOCX)
