# Supplementary material for: Wearable Sensor Use for Assessing Standing Balance and Walking Stability in People with Parkinson’s Disease: A Systematic Review
Source: PLoS One. 2015 Apr 20;10(4):e0123705. doi: 10.1371/journal.pone.0123705 (PMC4403989; doi:10.1371/journal.pone.0123705)
Supplement: S1 File — (DOCX) [file pone.0123705.s001.docx]

**Research Question**: Can wearable sensors be used to measure postural stability in people with Parkinson’s disease?

**Research Protocol:**

*Methods for Literature Search:*

A targeted search was conducted on January 30, 2015 of relevant databases for articles that were published within the past 20 years (1994-2014) and reported using wearable sensors to assess elements of standing balance or mobility in people with Parkinson’s disease (PD). Specifically, the databases searched were:

Pubmed

EMBASE

The Cochrane Library

Additionally, the bibliographies of the studies that met the inclusion criteria for this review were screened for relevant articles that may have been missed during the initial database searches. As potential papers were identified, they were added to an Endnote database to eliminate duplicate entries of research studies. The following outlines the complete combination of search terms that was used to search the titles and abstracts of potential papers for each of the three databases:

((((Parkinson's[Title/Abstract]) OR Parkinson[Title/Abstract]))

AND

((((Walk[Title/Abstract]) OR Gait[Title/Abstract]) OR Balance[Title/Abstract]) OR Stability[Title/Abstract]))

AND

(((((Acceleration[Title/Abstract]) OR Accelerometer[Title/Abstract]) OR Gyroscope[Title/Abstract]) OR Inertial[Title/Abstract]) OR Sensor[Title/Abstract])

*Strict Inclusion/Exclusion Criteria:*

To be eligible for inclusion in the systematic review, papers were required to meet the following inclusion and exclusion criteria:

*Inclusion Criteria:* For inclusion, papers were required to; i) involve a PD population; ii) utilise a body-mounted wearable sensor; iii) present at least one outcome measure for balance or postural stability during standing or walking; iv) be written in English; v) include a control group or control condition (e.g. ON vs. OFF medication); or vi) be a full-text article (i.e. not a conference abstract, systematic review or meta-analysis).

*Exclusion Criteria:* Papers were excluded if they had; i) no control group or control condition; ii) a mixed neurological participant sample; iii), no blinding to intervention status (if applicable); or iv) a wearable sensor that was a pedometer.

*Paper Review Process:*

A minimum of 2 reviewers (RPH and MHC) performed the initial screening of articles based on the title and abstract of the papers identified in the initial search and where discrepancies existed between the reviewers, they were discussed until a consensus was reached. The full-text of those papers that were considered potentially relevant following title and abstract screening were reviewed by 1 of the reviewers and papers that were eligible were subjected to quality assessment and data extraction (RPH). Where there were uncertainties about the relevance of a paper in the full-text review process, the second reviewer (MHC) was asked to independently evaluate the study and the inclusion status of the paper was discussed until a final consensus was reached.

*Quality Assessment:*

The methodological quality of each included paper was assessed using a previously-developed checklist described by Downs & Black (1998). This quality assessment checklist uses 27 questions to assess the reporting of external validity, bias and other potentially confounding factors that may have influenced the design and/or results of the study. For 25 of the criteria on the checklist, 1 point was awarded if the criterion was met by the paper and a score of zero was awarded if the criterion was not reported or could not be easily determined. One criterion concerning the description of potential confounders was assessed on a 2-point scale, where 2 points were awarded if this criterion was fully met, 1 point was awarded if it was partially met and no points were awarded if the information was not provided. Lastly, the criterion related to the reporting of a power calculation was assessed on a 5-point scale due to its increased importance for sample size justification. The sum of the scores for each of these items was divided by the maximum possible score for the assessment (32 points) and multiplied by 100 to yield a percentage that represented the manuscript’s methodological quality. Manuscripts were classified as having either very low (<25%), low (<50%, but ≥25%), moderate (<75%, but ≥50%) or high (≥75%) methodological quality.

*Downs, S. H., & Black, N. (1998). The feasibility of creating a checklist for the assessment of the methodological quality both of randomised and non-randomised studies of health care interventions. Journal of epidemiology and community health, 52(6), 377-384.*

*Methods for Data Extraction and Analysis:*

The initial step for this process involved a simple descriptive evaluation of each of the studies included in this review, which is presented in Table 1 of the manuscript. Furthermore, this table included a number of important pieces of information that were extracted from these studies and included:

Demographics – Experimental groups, disease severity, disease duration

Intervention – Description of intervention (if applicable)

Sensor Details – Type and placement

Postural Stability – Measures and modality of assessment

Findings – Results of the study
